# Error-related potentials detection to enhance human-robot collaboration: a mini review

**DOI:** 10.3389/fnrgo.2026.1769098

**Published:** 2026-05-25

**Authors:** David Achanccaray, Aurélie Clodic, Raphaëlle N. Roy

**Affiliations:** 1 Fédération ENAC ISAE-SUPAERO ONERA, Université de Toulouse, Toulouse, France; 2 LAAS-CNRS, Université de Toulouse, CNRS, Toulouse, France

**Keywords:** adaptation, error-related potentials, human-robot collaboration, human-robot interaction, passive brain-computer interface

## Abstract

Error-related potentials (ErrPs) have been studied to evaluate wrong decisions or actions in several contexts. An ErrP is an electrical potential on the scalp generated by the perception of errors and occurs unwittingly. In human-robot collaboration (HRC), ErrP detection can be used to trigger a feedback or an action to adapt the system to the user. This contributes to the improvement of HRC, taking into account user performance. However, to our knowledge, the detection of ErrPs in HRC has not been widely explored, resulting in only a few studies. This systematic review will present work on ErrP-based interfaces related to adaptation, control, and neuroergonomics for HRC. Thirteen articles were included after the exclusion criteria of the review stages. The average accuracy of ErrP detection was between 54 and 87.2%. In most cases, the authors simulated the occurrence of unexpected behavior of the robot. The robot mistakes occurred randomly between 20 and 35% of the total trials. Some works focused on the robot learning process and adaptation between humans and robots. The mental model and the robot behavior policy were updated based on the decoded ErrPs during collaborative interactions. Control-related works have included ErrPs detection/features as input inside the control loop or algorithm. Other studies assessed the influence of mental workload variability in the adaptation process, given that a high mental workload affects the cognitive processes needed to perceive errors. Thus, ErrPs present advantages for enhancing HRC, and this review opens the way to further developments in the robotic domain.

## 1 Introduction

Human-robot interaction (HRI) occurs in diverse application areas such as search and rescue, assistive robotics, military, education, entertainment, space, home, and industry (Goodrich and Schultz, 2008). Effective collaboration requires that robots adapt to human behavior, cognition, and emotions to optimize teamwork (Robert et al., 2020). In human-robot collaboration (HRC), humans and robots share goals and workspaces, coordinating either through physical contact (e.g., forces) or communication (e.g., gestures, voice, gaze, facial expressions). Robust multimodal communication is key to ensuring reliable and resilient collaboration (Mukherjee et al., 2022; Ajoudani et al., 2018).

Identifying errors resulting from wrong decisions or actions can benefit the adaptation process. Error detection can activate feedback, trigger an action, or be part of the robot's control algorithm. Error events evoke neural responses, which are called error-related potentials (ErrPs) when recorded from the scalp using an electroencephalogram (EEG). ErrPs are generated by the perception of errors and occur unintentionally. This perception of errors can occur for errors that are committed by the user themselves (they realize this themselves or are made aware via a given feedback), the system with which it interacts and its environment, or by another agent [human, robot or even artificial intelligence (AI)]. These brain potentials can be decoded with a passive brain-computer interface (BCI). A passive BCI monitors brain activity while the user does not control it voluntarily; however, the user has an active role during the human-machine interaction. This determines their engagement level, which can in turn affect error production and perception, and therefore ErrP occurrence (Chavarriaga et al., 2014; Yasemin et al., 2023).

ErrP detection has shown potential to improve adaptation between humans and intelligent systems (Klaproth et al., 2024). However, to our knowledge ErrP detection has not yet been widely explored in HRC, resulting in only a few available studies. In this review, we focused on identifying applications where ErrP detection improved HRC. This review did not consider articles that only aimed at modeling decoders for ErrP detection. This survey answered the following research questions (RQs):

- RQ 1—What type of error is used depending on the HRC application?
- RQ 2—What type of HRC configurations can be improved with an ErrP-based passive BCI?
- RQ 3—What approaches can be used to enhance HRC with an ErrP-based passive BCI?

## 2 Background

This section will review research fields related to ErrPs and HRC to prepare the basis for this review.

### 2.1 Error-related potentials

**Figure 1:**
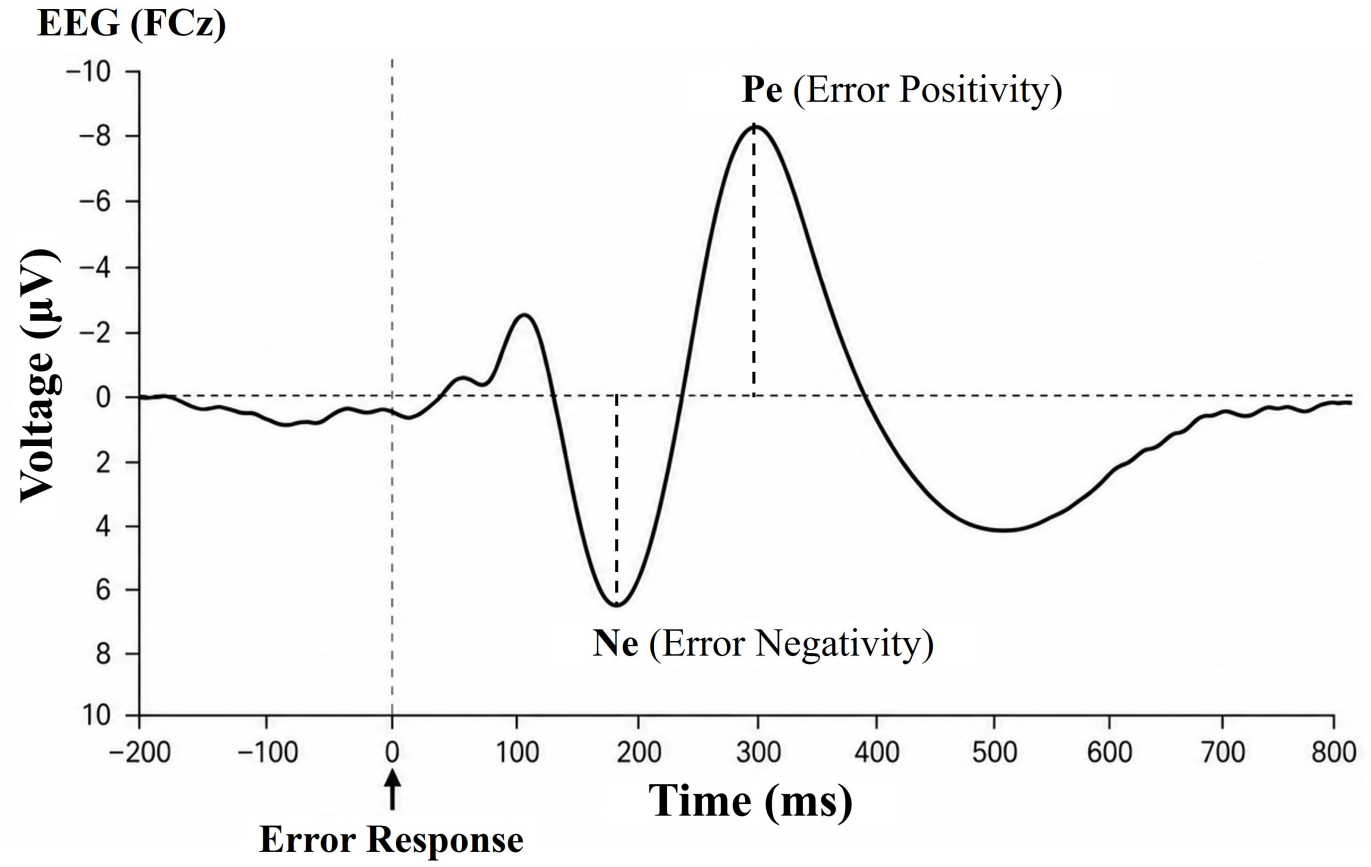
Grand average of ErrP signals from EEG at FCz electrode.

ErrPs were characterized in the early 90s. This event-related potential showed a negative peak (error-related negativity) in the fronto-central cortex around 80 ms after the error occurrence in a choice task. A larger positive component in the centro-parietal cortex follows the negative peak in the time window of 200–500 ms (Figure 1) (Falkenstein et al., 1991; Gehring et al., 1993; Falkenstein et al., 2000). The error occurrence also elicited frequency modulations, such as an increase in the theta band (4–8 Hz) followed by a decrease in the beta band (12–30 Hz). In addition, connectivity analysis revealed the influence of the anterior cingulate cortex on the prefrontal cortex. A similar response (feedback-related negativity) is generated when feedback is shown to the user to indicate wrong performance and appears in the time window of 200–300 ms after the feedback presentation (Chavarriaga et al., 2014; Yasemin et al., 2023; Iwane et al., 2023b).

### 2.2 Human-robot collaboration

Robots and humans work together as a team in HRC. They share a workspace and task goals while carrying out parallel tasks. Collaboration can involve physical contact through exchange forces or be contactless through gestures, voice commands, motion intentions, facial emotions, or eye gaze to achieve effective communication. Multimodal communication makes the system redundant and more robust to unexpected noise and perturbations. Thus, Robot learning strategies are defined by training machine learning models using collected multimodal data (Mukherjee et al., 2022; Ajoudani et al., 2018). Machine learning techniques in HRC can be grouped as follows: unsupervised learning, reinforcement learning (RL), and supervised learning. They include classical models [linear discriminant analysis (LDA), support vector machine (SVM),...] and deep learning models [convolutional neural networks (CNNs),...]. These techniques have been applied to different collaborative tasks, such as collaborative assembly, object handover, object handling, and collaborative manufacturing. Collaborative tasks can be evaluated using metrics categorized as precision of robot movement, robustness, proof of concept, performance improvement, and reduction of physical workload (Semeraro et al., 2023).

HRC reduces human workload by optimizing the strengths of humans and robots in a specific task. Physiological computing can enhance HRC systems. The robot accesses the human physiological response information and interprets the human state, which can increase awareness within workplaces and HRC efficiency (Roy et al., 2020). Physiological computing can be provided from multimodal sources, such as EEG, electrocardiogram, electrooculogram (EOG), electromyogram (EMG), electrodermal activity, skin temperature, respiration, and eye-trackers. Physiological computing has been used for different purposes, such as control, mental state assessment, stress level detection, and emotion recognition (Rückert et al., 2023). Stress level, workload and fatigue are relevant mental states to try and take into account during HRC, especially under demanding conditions (Achanccaray et al., 2025). Automation can generate new stressors for humans by reducing task control and social interaction, and increasing work pace and psychological pressure due to performance monitoring. The complexity of HRC can also increase mental workload, and robot efficiency could cause concern about job security among workers. Another issue is that the robot's proximity to the workers can generate fear because it is potentially harmful. However, collaborative robots can alleviate humans from performing heavy or dangerous tasks, in order to achieve optimal levels of mental workload and improve task performance (Carissoli et al., 2024). It should be noted that mental workload is affected by robot-related factors, such as robot features (dimensions and speed), social touching (human-like communication and interaction), and robot movement trajectory (unpredictable possible damage). These factors depend on human perception, which influences the evaluation metrics of collaborative tasks (Lu et al., 2022).

### 2.3 BCIs based on error-related potentials

The most used application of BCI that measure and incorporate ErrPs are spellers, where ErrPs increase P300-based BCI performance (Spüler et al., 2012; Cruz et al., 2018; Zeyl et al., 2016). ErrPs detection has been used in other applications, such as cursor control (Schalk et al., 2000; Ferrez and Millan, 2008), rehabilitation (Kumar et al., 2019, 2021; Soriano-Segura et al., 2024), HRI (Ehrlich and Cheng, 2019b; Salazar-Gomez et al., 2017; Wang et al., 2023; Ciabattoni et al., 2019; Ehrlich and Cheng, 2016; Perrin et al., 2010; Iwane et al., 2023a; Fava et al., 2024), adaptation in HRC (Ehrlich and Cheng, 2018, 2019a; Kim et al., 2017, 2020a,b; DelPreto et al., 2020; Buerkle et al., 2021), control in HRC (Ferracuti et al., 2021; Omer et al., 2025; Schiatti et al., 2018; Aldini et al., 2021), neuroergonomics in HRC (John et al., 2024; Fuji and Natsume, 2024), and ErrPs decodification in HRI (Lopes-Dias et al., 2019; Kim et al., 2023; Omer et al., 2022; Singh et al., 2020). In this review, we focus on HRC applications, with works on adaptation, control, and neuroergonomics. These studies are detailed in section 4. We did not include works on the decodification of ErrPs because they are machine-learning articles.

## 3 Methods

**Figure 2:**
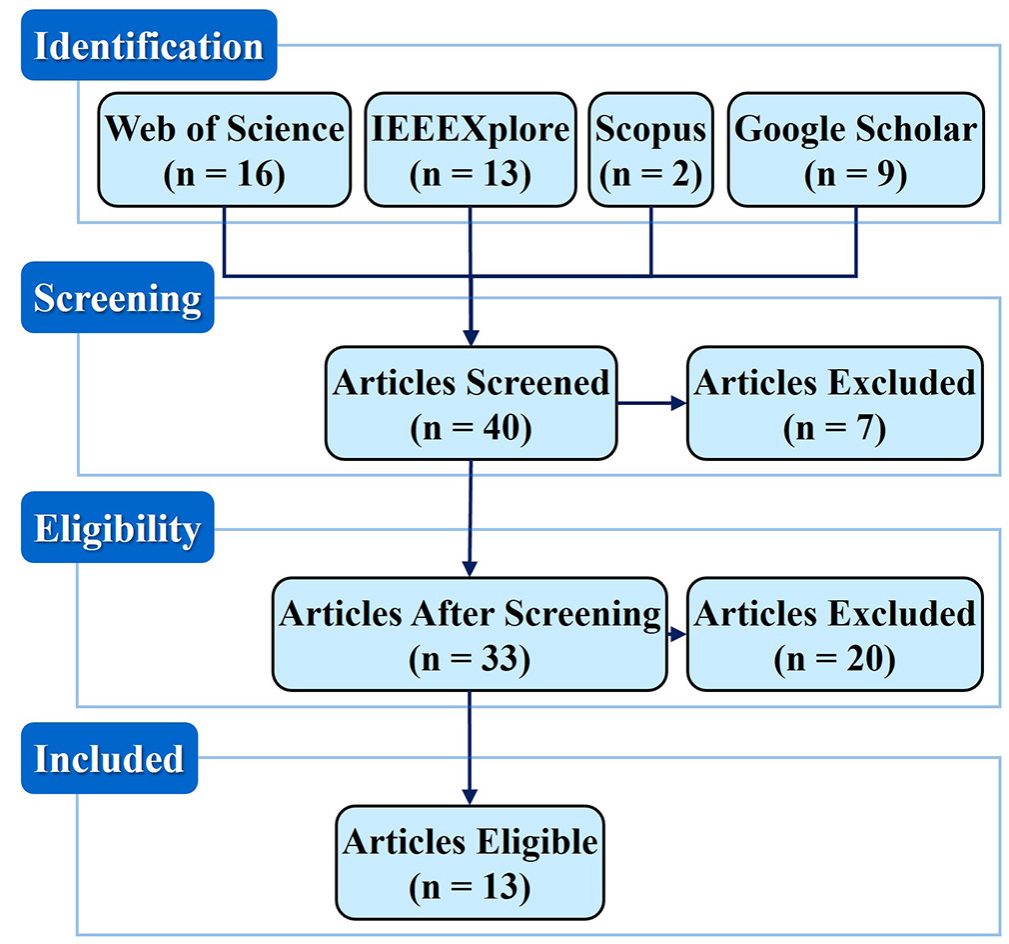
Flow chart of the systematic review process.

In the following section, we will detail the methodology adopted–PRISMA protocol (Tricco et al., 2018)–to elaborate this systematic review to answer our research questions. Figure 2 shows the flow chart of the systematic review process.

- Identification: the search was conducted in October 2025 in four databases: Web of science, IEEEXplore, Scopus, and Google Scholar. They are the most commonly used to cover the different technological areas involved with this review. The search scope was limited only to published articles. Keywords related to HRI, BCI, and HRC features were grouped into three sets (Table 1). The three sets were combined using the Boolean logic operation “AND” between each set and the Boolean logic operation “OR” within each set. Articles only aimed at modeling decoders for ErrP detection were excluded. This search resulted in a total of 39 publications.
- Screening: in this stage, all articles were examined by reading the title and abstract. The article's content was also examined when the abstract was not well-detailed. The criteria for including articles in the next stage were as follows. Articles should be written in English, not review or survey papers, mainly related to HRC, and listed only once.
- Eligibility: the whole content of each article was examined in this stage. The criteria for including articles in the next stage were as follows. Articles should be written in English, not review or survey papers, mainly related to HRC, and available in full text.
- Included: parameters were extracted from the included articles to be shown in the next section 4. The parameters were the following: type of error to elicit ErrPs, HRC configuration, ErrP-Based BCI metrics, and Collaborative task metrics.

**Table 1:** Keywords used in the systematic search.

HRI	(AND) BCI	(AND) HRC features
“human-robot interaction” OR “human-robot collaboration” OR “human-robot cooperation”	“error potential” OR “error-related potential” OR “error-related potentials”	“adaptation” OR “coadaptation” OR “human-centered AI” OR “control”

## 4 Results

The total number of articles found was 40. After the exclusion criteria in each stage, the articles included in this review were 13. These results were published in the last 9 years, and reflect the relevance of ErrPs in HRC in recent years. However, all the results were developed in laboratory environments. They are summarized in Table 2. This section presents these articles into three categories: adaptation in HRC, control in HRC, and neuroergonomics in HRC.

**Table 2:** Summary of articles found in this review.

Categories	Context	Task	Trigger	Details	Setup	ErrPs Detector	References
Adaptation	Gaming	The robot chose one of three objects. The human guessed the robot's gaze pattern	Wrong human guess ⇒ Updated the human and robot behavior policies	Improvement by ErrP ≈34%–57%	EEG (27 Ch), EOG (3 Ch), Humanoid Robot NAO	(Online) Regularized LDA	Ehrlich and Cheng, 2018, 2019a
	Robot learning	The human interacted with the robot through gestures. The robot should associate these gestures with actions. No previous associations	ErrP detection ⇒ Reward in RL = ErrP	Actions ⇔ Gestures ↑ Learning	EEG (64 Ch), Leap Motion Controller, Robot arm COMPI, MARS^d^ simulator	(Online) SVM	Kim et al., 2017
			Robot actions ≠ gestures ⇒ Updated the robot's behavior policy	ErrP Detection 87.2% (H^a^) 86.9% (R^a^)			Kim et al., 2020a,b
	Human supervision of robots	Multiple-choice robot tasks. Robot selected targets for a drilling operation	ErrP detection ⇒ Human hand-gestures to correct the robot's choice	EMG and EEG framework feasibility	EEG (48 Ch), EMG (NI USB-6216 2 Ch), Robot arm Baxter	(Online) Feed-forward neural networks	DelPreto et al., 2020
		Unexpected events (crush, drop, unexpected movement) in assembly robot tasks	ErrP detection ⇒ Robot stop interface	ErrP detection emergency stop feasibility	EEG (14 Ch), Robot arm UR10	(Offline) Decision tree	Buerkle et al., 2021
Control	Path planning	The human defined the target of a virtual smart wheelchair. The control algorithm determined the path planning	ErrP detection ⇒ Recalculated path planning	ErrP detection complemented the navigation sensors	EEG (8 Ch), Smart wheelchair Quickie Salsa R2, Gazebo^d^ simulator	(Offline) Bayesian LDA	Ferracuti et al., 2021
		Comparison between SSVEP^c^ and (Ferracuti et al., 2021) approaches to correct the path planning	ErrPs: ↑ Task achievement ↓ Engagement		EEG (8 Ch), Wheelchair-mobile robot, Gazebo^d^ simulator	(Online) Bayesian LDA	Omer et al., 2025
	Robot learning	Robotic arm moving in a mono-dimensional grid to a target on a screen following a trajectory	Erroneous robot behavior ⇒ ErrP was an input of the RL training	ErrP reduced variability of RL training	EEG (16 Ch), Robot arm UR5	(Online) SVM	Schiatti et al., 2018
	Complex control	The human moved the robot's end- effector through its workspace	Singularity positions ⇒ Generate ErrP	ErrP related to lack of predictability	EEG (32 Ch), Robot arm UR10	(Offline) Signal processing	Aldini et al., 2021
Neuro- ergonomics	Cognitive workload	Control of a robot arm end- effector and a mental calculation	Unexpected stop of the robot movement ⇒ Generate ErrP	↑ Mental workload ⇒↓ Error awareness	EEG (32 Ch), Robot arm UR10	(Offline) EEGNet^b^, ShallowConvNet^b^, DeepConvNet^b^	John et al., 2024
	Boredom	Robot exploration task to reach a target in a large-scale virtual maze	Wrong robot behavior (di- rection) ⇒ Generate ErrP	↑ Boredom ⇒↓ ErrPs amplitude	EEG (7 Ch), Gazebo^d^ simulator	(Offline) CNN	Fuji and Natsume, 2024

^a^H, Human perspective; R, Robot perspective.

^b^EEGNet, ShallowConvNet, and DeepConvNet are state-of-the-art CNNs.

^c^SSVEP, Steady-state visually evoked potentials.

^d^MARS simulator: http://mars-sim.org, Gazebo: https://gazebosim.org.

### 4.1 Adaptation in HRC

Six articles were found in this category. In a gaming context (Ehrlich and Cheng, 2018, 2019a), the robot chose one of three objects, and the human had to guess the robot's gaze pattern. The human and robot's behavior policies were updated whenever the human's guess was incorrect. This adaptation was carried out using the ErrP decoded with an average accuracy of ErrP detection of around 81.8%. In addition, the average percentage of right guesses increased from 33% to 70%–90% within 10–40 trials.

Other studies were focused on robot learning, where the human interacted with the robot through gestures (Kim et al., 2017). The robot learned to associate actions to non-predefined gestures during the HRI by using the ErrPs as reward in the interactive RL algorithm. This study was extended to adapt the learning policy to online changes of human intent by adding new gestures (Kim et al., 2020a,b). The learning algorithm recognized human gestures and triggered a robot action. If this action did not correspond to the gesture, the evoked ErrP was used to update the robot's behavior policy. The performance of the ErrP detection depended on the human or robot perspectives. The ErrP detection accuracies reached 87.2 and 86.9% for the human and robot perspectives, respectively. This analysis revealed a high correlation (ρ) between robot's learning performance and ErrP detection performance. The ρ values were between 0.897 and 0.934 (*p* < 0.01) for the human perspective, and between 0.869 and 0.965 (*p* < 0.01) for the robot perspective.

Another application is the human supervision of robots to correct mistakes during multiple-choice tasks (DelPreto et al., 2020). In this study, the autonomous robot selected targets for a drilling operation. The authors processed multimodal signals (EMG and EEG). ErrP detection triggered the correction sequence and continuous pattern recognition of EMG signals from left/right hand-gestures of the human supervisor to correct the robot's choice. This hybrid framework reached the 97.3% of correct targets, an average accuracy of around 54% of ErrP detection (tested with only two participants), while the average accuracy of EMG classification (tested with only three participants) for the left hand-gestures was of 65.8% and right hand-gestures was of 85.2%. The EEG signal classification should be improved to ensure a reliable hybrid system. However, two error detection systems working in parallel resulted in an almost perfect performance for this task. In addition, the feasibility of using ErrP to detect potential emergencies in HRC in a manufacturing context was approached in Buerkle et al. (2021). The emergencies were generated by simulating errors in the robot's movement execution in an assembly task. There were three object manipulation cases: crush, drop, and unexpected movement. This study found an optimal time window of 250 ms to process EEG signals. However, the participants had no experience in HRI tasks, which contrasted with real robot operators. This experience difference influences the neural response evoked in emergencies.

### 4.2 Control in HRC

Four articles were found in this category. A human-in-the-loop approach was applied to correct the path planning of assistive mobile robots in a virtual scenario (Ferracuti et al., 2021). The user controlled a smart wheelchair. The robot control algorithm determined the path planning to reach the target defined by the user. The path was recalculated when an ErrP was evoked by the approach of the robot to the obstacles. The overall ErrP detection accuracy was 78.6%. This study was extended to evaluate the most effective human feedback integration (Omer et al., 2025). ErrPs and steady-state visually evoked potentials (SSVEP) approaches were compared, where the ErrPs approach reached higher task achievement and lower engagement than the SSVEPs approach.

In addition, human in the loop of robot learning was based on ErrPs detection in Schiatti et al. (2018). ErrP detection evoked by the erroneous robot behavior was an input to update the reward in the RL algorithm during the training stage. This study included feedback conditions, such as visual and visuo-tactile feedback. The ErrP detection average accuracies were 60 and 59% for visual and visuo-tactile feedback, respectively. RL for route learning showed convergence at around nine iterations for both feedback conditions. However, the number of iterations was lower for visuo-tactile feedback than for visual feedback. Regarding more complex control methods, singularity avoidance strategies with complex dynamics in HRC generated detectable ErrP (Aldini et al., 2021). This study reported that the participants preferred the strategy that produced greater robot predictability (faster response), which reflected a lower power spectral density of the ErrPs.

### 4.3 Neuroergonomics in HRC

Two articles were found in this category. In the first, cognitive workload affected the error awareness of a user in HRC (John et al., 2024). This resulted in reduced ErrPs. In this study, the participants evaluated two different workload conditions by performing two tasks. The primary task consisted of interacting with a game by controlling a robot arm end-effector of two degrees of freedom. The secondary task was a mental calculation with varying difficulty. ErrPs were generated by unexpected stops of the robot movement in 33% of the trials. A high mental workload mitigated the ErrP amplitude, which evidenced a reduced error awareness. In addition, there was an increase in the theta band power in the frontal cortex and a trend to increase the power of alpha and beta bands in the central cortex after the unexpected stop, compared to the regular stop when the robot reached its target.

The second study focused on boredom, which negatively influences the performance of collaborative tasks when user engagement is reduced (Fuji and Natsume, 2024). This work assessed the mental state of participants while they performed an exploration task to reach a target in a large-scale maze in collaboration with a robot in a simulated environment. The robot was autonomous, but it consulted with participants about the direction it should take at intersections. The robot was programmed to change direction in 30% of the trials and generated ErrPs. There was an increase in the theta band power and a decrease in the beta band power. In the second half of the task, some participants felt bored, which decreased the ErrP amplitude and the theta band power.

## 5 Discussion

Regarding ErrPs in HRC studies, an unexpected robot behavior generated errors caused by wrong signal processing or being out of the workspace scope of the robot's sensors. These errors were independent of the application. The average accuracy of ErrP detection was between 54 and 87.2%. In most cases, the robot's mistake was programmed to occur randomly between 20 and 35% of the total trials (DelPreto et al., 2020; Buerkle et al., 2021; Ferracuti et al., 2021; Omer et al., 2025; Schiatti et al., 2018; John et al., 2024; Fuji and Natsume, 2024). In the rest of the cases, the unexpected action occurred naturally during the robot's learning process, or the robot's interaction with the environment in a collaborative task (Ehrlich and Cheng, 2018, 2019a; Kim et al., 2017, 2020a,b; Aldini et al., 2021). Although it is easier to provoke or program the robot's error, the error's natural occurrence would be closer to a real case. Usually, the robot's autonomy algorithm is trained to be robust and avoid errors. However, natural errors can occur during the robot's learning in the adaptation stage. It would depend on the experimental design and the application. Thus, it answered the first research question about the type of error.

The second research question was about the HRC configuration. HRC studies used ErrPs in several tasks, such as games (Ehrlich and Cheng, 2018, 2019a; John et al., 2024), gesture learning (Kim et al., 2017, 2020a,b), supervision (DelPreto et al., 2020), assembly (Buerkle et al., 2021), navigation (Ferracuti et al., 2021; Omer et al., 2025; Fuji and Natsume, 2024), and control (Schiatti et al., 2018; Aldini et al., 2021). These tasks involved physical and contactless interactions between the human and the robot. The robots were humanoid robots, robot arms, or mobile robots. ErrPs were evoked by visualizing the unexpected robot behavior. In most cases, adaptation was approached in HRC, which was configured as a contactless interaction or manipulation of an object with humanoid robots or robot arms. In these last HRC applications, the system's AI updated robot behavior policies based on ErrP detection.

An interesting approach for real applications that answered the third research question could be contactless interaction in an exploration or search task, or manipulating an object as an assembly task. These scenarios will provide conditions for the natural occurrence of unexpected robot behavior. An ErrP-based passive BCI would detect these robot errors to enhance adaptation in HRC during the robot learning process in the human-centered AI. In addition, safety must be guaranteed when deploying these types of systems. Thus, the use of wireless EEG is necessary. This device must remain fixed to avoid any potential interference with the workspace. This approach gives us a wide spectrum of options to improve HRC.
